# Relationships between Irritable Bowel Syndrome Pain, Skin Temperature Indices of Autonomic Dysregulation, and Sensitivity to Thermal Cutaneous Stimulation

**DOI:** 10.1155/2010/949027

**Published:** 2010-08-09

**Authors:** Fong Wong, Anthony C. Rodrigues, Christopher D. King, Joseph L. Riley, Siegfried Schmidt, Charles J. Vierck, Andre P. Mauderli

**Affiliations:** ^1^Department of Prosthodontics, College of Dentistry, University of Florida, 1600 SW Archer Rd, D11-006 Gainesville, FL 32610, USA; ^2^Tufts University School of Medicine, Boston, MA 02111, USA; ^3^Department of Community Dentistry and Behavioral Science, College of Dentistry, University of Florida, 1600 SW Archer Rd, D11-006 Gainesville, FL 32610, USA; ^4^Community Health & Family Medicine, University of Florida, Gainesville, FL 32610, USA; ^5^Department of Neuroscience, College of Medicine, University of Florida, Gainesville, FL 32610, USA

## Abstract

This study evaluated relationships between irritable bowel syndrome (IBS) pain, sympathetic dysregulation, and thermal pain sensitivity. Eight female patients with diarrhea-predominant IBS and ten healthy female controls were tested for sensitivity to thermal stimulation of the left palm. A new method of response-dependent thermal stimulation was used to maintain pain intensity at a predetermined level (35%) by adjusting thermal stimulus intensity as a function of pain ratings. Clinical pain levels were assessed prior to each testing session. Skin temperatures were recorded before and after pain sensitivity testing. The temperature of palmar skin dropped (1.5°C) when the corresponding location on the opposite hand of control subjects was subjected to prolonged thermal stimulation, but this response was absent for IBS pain patients. The patients also required significantly lower stimulus temperatures than controls to maintain a 35% pain rating. Baseline skin temperatures of patients were significantly correlated with thermode temperatures required to maintain 35% pain ratings. IBS pain intensity was not significantly correlated with skin temperature or pain sensitivity. The method of response-dependent stimulation revealed thermal hyperalgesia and increased sympathetic tone for chronic pain patients, relative to controls. Similarly, a significant correlation between resting skin temperatures and thermal pain sensitivity for IBS but not control subjects indicates that tonic sympathetic activation and a thermal hyperalgesia were generated by the chronic presence of visceral pain. However, lack of a significant relationship between sympathetic tone and ratings of IBS pain casts doubt on propositions that the magnitude of IBS pain is determined by psychological stress.

## 1. Introduction

Irritable bowel syndrome (IBS) is a functional pain disorder with no known etiology but an association with psychological stress [1–3]. Because autonomic output systems of central stress circuits control essential functions of the gut [4–6], stress has been suspect as a cause of IBS [1, 5, 7]. Levels of stress can be revealed as increased sympathetic and/or decreased parasympathetic activity, and IBS patients often have enhanced sympathetic activation [8–14] or reduced parasympathetic activation [8, 14–19] and/or an increased sympathetic/parasympathetic ratio [14, 17, 19, 20]. Numerous factors can change relationships between diagnoses of IBS and levels of autonomic activation including age, duration of chronic pain, gender, profiles of gut disturbance (e.g., abdominal pain with constipation or diarrhea), magnitude of IBS pain, skin temperature, and the method of autonomic stimulation. 

Correlations between autonomic dysregulation and IBS should not be construed to establish stress as a cause of abdominal pain, because pain is a potent source of stress and therefore of sympathetic activation [10, 21–23], which can increase pain sensitivity [10, 24, 25]. Thus, pain from pathological processing within the gut generates stress, and stress can increase pain produced by stimulation of the gut [5, 26, 27], setting up a vicious cycle. Relationships between the presence of pain and autonomic dysfunction in IBS patients have been shown in models that used blood pressure, heart rate variability, and heart rate as indicators [10, 28, 29]. Visceral (rectal distention) and repeated large area somatic (foot immersion in a hot water bath) stimuli were used to evoke the responses. In the present study, we hypothesize that abnormal autonomic responsivity (skin thermoregulation) can be revealed by prolonged focal thermocutaneous stimulation. Several minutes long episodes of thermocutaneous pain were induced with a new stimulation method that makes the procedure safe and tolerable for the subjects [30].

We evaluated clinical pain levels of female IBS patients with diarrhea. IBS is more prevalent among females than males [31], and females are particularly susceptible to stress-related pain conditions [32]. Females have been characterized in terms of resting parasympathetic dominance, compared to males [33], and according to some studies IBS patients with diarrhea are especially prone to sympathetic dysregulation [10, 34–37]. These characteristics should maximize detection of sympathetic activation for comparison with levels of IBS pain. Skin temperature was selected as a sensitive measure of sympathetic activation for females [13, 25, 38, 39]. Painful thermal stimulation was chosen as a method of phasic autonomic activation, because the impact of this procedure could be quantified as the magnitude of elicited pain, which could be related to skin temperatures. 

## 2. Methods

### 2.1. Subjects

The subjects were eight female IBS patients that were diagnosed with diarrhea-predominant IBS (age range of 21–53; mean age of 36.3 years) and ten healthy female controls (age range of 19–45; mean age of 27.0 years). Written informed consent was obtained from all participants once the nature of the study had been thoroughly explained. The procedures were conducted under approval of the University of Florida Institutional Review Board and the Veterans Administration SCI committee. 

The criteria for members of the control group required no significant spontaneous pain anywhere in the body, no ongoing pharmacotherapy with narcotics or antidepressants, and no disease that might significantly affect pain perception or unduly increase risk of injury (e.g., neurological disorders, serious psychiatric disorders, diabetes, hypertension, serious cardiovascular disorders, and chronic pain diseases such as fibromyalgia syndrome). The criteria for the disease group required a diagnosis of ongoing IBS based upon the Rome II criteria [40], supplemented by additional criteria—absence of other diseases (including other chronic pain diseases), risk factors, and ongoing drug treatments—as described for the control group. Patients with any condition where spontaneous pain was widespread, according to the definition of the American College of Rheumatology [41], were excluded from the study. This ruled out participation of subjects that met the ACR diagnostic criteria for fibromyalgia syndrome. Initial screening consisted of blood pressure measurement, completion of a health questionnaire, and for IBS patients, clinical diagnosis by a physician. All of the patients reported recent and recurring episodes of spontaneous abdominal pain or discomfort. All subjects were right-handed. 

### 2.2. Response-Dependent Thermal Stimulation

The subjects were asked to rate pain intensity continuously by making adjustments to the slider of an electronic visual analog scale (eVAS). Instructions regarding the use of the scale and its end points (“no pain” and “intolerably intense pain”) were given by a standardized video. The slider's position was recorded as a percentage of its total travel. The eVAS was mounted into the surface of a small inclined desk, which was positioned to facilitate precise operation with minimal fatigue. During the entire experiment, the subject was separated from the investigator by an equipment rack and was facing away to minimize nonverbal communication and transmission of bias. 

Thermal stimuli were administered with a flat copper contact thermode 23 × 23 mm in size. The thermode was electronically held at the desired temperature by a Peltier thermoelectric device. It was brought into light skin contact of reproducible force by solenoid activation. Control software for the stimulator sampled pain intensity ratings to make automatic adjustments in thermode temperature to maintain an average pain rating that equaled a set point of 35% (35 on a VAS scale of 0–100). The dependent variable for response-dependent stimulation was the thermode temperature, and high pain sensitivity was revealed by a low average thermode temperature [30].

**Figure 1:**
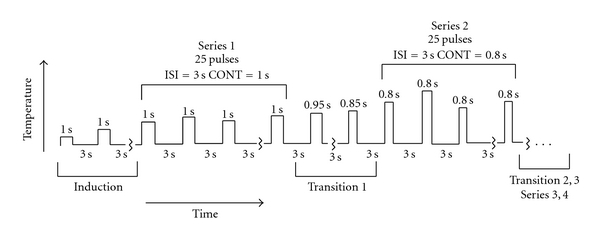
The stimulus duration alternated between 1.0 second (series 1 and 3) and 0.8 seconds (series 2 and 4). Within each series, the temperature was modulated in a pain rating-dependent manner to maintain average pain intensity near a predetermined setpoint (35%). The change in temperature needed to compensate for the change in stimulus duration or ISI served as response variable. Stimulus durations are indicated above each bar, and interstimulus intervals remained constant at 3.0 seconds.

 Four series of 25 brief thermal contact pulses each were delivered to the palm of the left hand (glabrous skin of the thenar eminence) in 3 separate testing sessions on different days. The interval between stimuli was 3 seconds, with stimulus duration (SD) of 1.0 seconds for series 1 and 3 and 0.8 seconds for series 2 and 4. The SD was transitioned across 4 pulses using increases or decreases of 0.4 seconds between series (Figure 1). 

Stimulation began with a 43°C pulse, which was never perceived as painful by any subject. The temperature then increased from pulse to pulse in 1°C increments until pain intensity reached 10% on the electronic visual analog scale. Thereafter, the temperature continued to rise at a reduced rate (0.5°C/pulse). This induction phase ended when the pain intensity rating first reached or exceeded the set point of 35% on the eVAS. At that point bidirectional temperature modulation commenced, maintaining a constant average pain intensity level for the remainder of the series.

### 2.3. Measurement of Spontaneous Pain

 At the beginning of all experimental sessions, subjects were asked to shade the locations and spatial extent of spontaneous pain on an anatomical diagram and to rank these sites according to pain intensity. Subsequently, the intensity of disease-related pain of the upper body (head, neck, shoulder, upper back, arms, and hands) and lower body (low back, bowel, legs, and feet) were rated with the eVAS. The subjects were then asked to rate the pain at the single most intense site. All these ratings were required to be below 5% (on the 0–100% eVAS scale) for subjects to be admitted to the control group.

### 2.4. Measurement of Skin Temperature

Skin temperatures at both the stimulation site (left) and the corresponding contralateral site (right) were measured with an Exergen Dermatemp infrared temperature scanner model DT-1001 (Exergen Corp., Watertown, MA, USA) prior to, immediately after, and 3 minutes following administration of the four stimulus series. The temperature was scanned for 1-2 seconds, and the highest temperature reading during this period was recorded. For testing associations between skin temperature and pain sensitivity, two variables were used; baseline temperature and maximum change in skin temperature at the nonstimulated palm, both averaged across three days of testing.

### 2.5. Statistical Methods

The GLM module of SPSS 17.0 was used to test hypotheses. Correlation coefficients between ratings of clinical pain, baseline skin temperature, maximum change in skin temperature at the nonstimulated palm, and average probe temperature across the series were calculated using Pearson Product Moment correlation coefficients. Because of the small samples involved, only correlations of over  .50 are reported.

Skin temperature differences between IBS patients and healthy controls at the stimulated and nonstimulated palms were tested using repeated measures analysis of variance (ANOVA). The same analysis was also used to test group differences in the stimulus temperatures needed to (a) first reach an eVAS rating of 10% and 35% and (b) maintain a rating of 35% for each series. Bartlett's test of sphericity of the residual covariance matrix was used to test the sphericity assumption. Greenhouse-Geisser adjustments were made where appropriate. Paired-samples *t*-test using *P* = .01 as the critical value was used for all pairwise comparisons.

## 3. Results

### 3.1. Ratings of IBS Pain

Ratings of the distribution and magnitude of ongoing pain were obtained prior to each testing session. Gut pain was rated as most severe on all but 1 occasion for 3 different IBS patients, when upper extremity pain was slightly greater than gut pain. Gut pain was rated on test day 1 at an average of 28.5% (SD = 16.8, range = 3–51), on day 2 at an average of 20.8% (SD = 23.9, range = 1–72), and on day 3 at an average of 22.2% (SD = 22.8, range = 5–61). Thus, ongoing pain can be characterized overall as mild to moderate, but it varied considerably from day to day across the group of IBS patients.

### 3.2. Skin Temperature

Skin temperature at the thenar eminence of both hands prior to thermal stimulation did not differ between IBS and control groups or between sessions. At the stimulated palm, the results of a group × day × time ANOVA for skin temperatures indicated a significant main effect of time (*F* = 165.973, *P* < .001). As expected, a higher mean temperature was found immediately following the stimulus application than at baseline or following the 3-minute recovery period (both at *P* < .001). The baseline and recovery temperatures were not different. There were no other significant main or interaction effects.  Table 1 presents the mean temperatures by site and time for IBS patients and controls, collapsed across days. 

**Figure 2:**
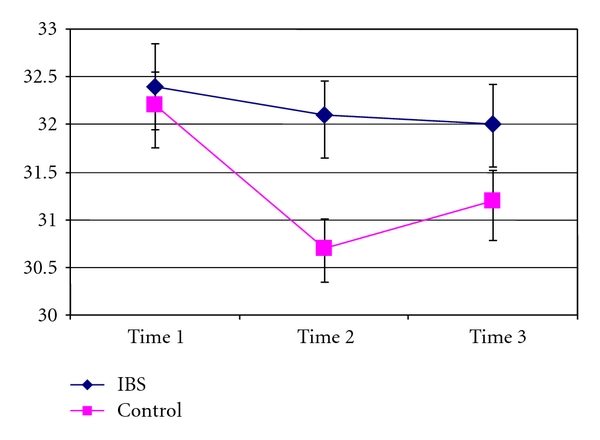
Non-tested palm. Average temperatures (ordinate) for the left and right palmar surfaces of control subjects and IBS patients at 3 time points (abscissa). Compared to the resting level (time 1), 5 minutes of pain testing for control subjects significantly reduced skin temperature (time 2), with some recovery by 3 minutes (time 3). Resting skin temperatures did not differ significantly for IBS and controls subjects. A significant decrease in skin temperature was not produced by pain testing of IBS patients.

At the unstimulated palm, there was a substantial drop in skin temperature that was detected immediately after thermal stimulation, with partial recovery 3 minutes later. There were significant main effects for group (*F* = 5.772, *P* = .029) and time (*F* = 9.513, *P* = .001) and a significant group by time interaction (*F* = 4.028, *P* = .028). Testing within groups to interpret the significant interaction, the unstimulated palmar temperatures for the control group were significantly different across times 1, 2, and 3 (*P* < .001). There were no significant differences across times 1–3 for the unstimulated palm of the IBS patients. These data are plotted in Figure 2.

### 3.3. Response-Dependent Evaluation of Thermal Pain Sensitivity

Thermode temperature differences associated with initial pain ratings of 10% and 35% for the control and IBS groups were tested using group × day ANOVA. Significant main effects of group (*F* = 5.8498, *P* = .028) and ratings (*F* (1, 16) = 30.687, *P* < .001) were found. Controls required a higher temperature to reach the pain rating criteria than the IBS group (Table 2). As expected, a higher mean temperature was associated with the rating of 35% compared to 10%.

**Figure 3:**
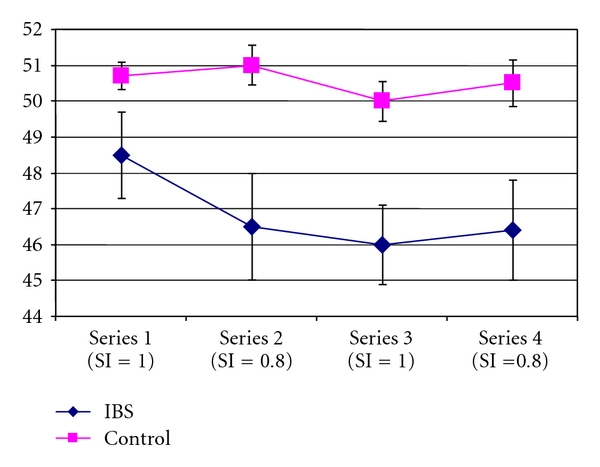
Mean thermode temperature by series. Thermode temperatures (ordinate) averaged across 3 sessions of response-dependent stimulation that maintained an average eVAS rating of 35 for pain intensity. Thermode temperatures are shown for 4 sequential series of 25 stimuli with durations of 1.0 second (series 1 and 3) and 0.8 seconds (series 2 and 4). Hyperalgesia for cutaneous thermal stimulation was revealed by lower thermode temperatures within each series.

The average thermode temperatures required to maintain a rating of 35% are shown in Figure 3 and Table 2 as a function of pulse duration. A group × day × series repeated measures ANOVA revealed a significant main effect of group (*F* = 9.592, *P* = .007), as well as a group × series interaction (*F* = 3.234, *P* = .022). The controls used higher temperatures for the 0.8 CI series compared to the 1.0 CI series as expected. Mean thermode temperatures differed for controls between series 2 and 3 (*P* < .001) and series 3 and 4 (*P* < .001). The IBS group sensitized after the first series, which differed from series 2 (*P* < .01), series 3 (*P* < .001), and series 4 (*P* < .01).

### 3.4. Relationships between Clinical Pain, Skin Temperatures, and Thermode Temperatures

For IBS patients, there were no significant associations between ratings of clinical pain at the most painful site on the day of testing and resting skin temperatures, skin temperatures at the unstimulated palm after thermal pain testing, or thermode temperatures that maintained 35% ratings. In contrast, thermode temperatures for the IBS group were associated with baseline skin temperatures (*r* = .74, *P* = .014) and with the maximum change in skin temperature on the nonstimulated palm (*r* = .52, *P* = .11). Resting skin temperatures, skin temperature changes during stimulation, and thermode temperatures were not significantly correlated among controls.

## 4. Discussion and Conclusions

The present study found that abnormal autonomic responses of IBS patients are revealed by painful somatic stimulation of a small area of the skin, as long as the stimulus is of sufficient duration. Limitations of the study are that the duration and intensity of the painful stimuli were not systematically varied, and traditional autonomic response measures (heart rate variability, blood pressure) were not included, for comparison.

A repeated measures design was utilized for evaluation of clinical pain ratings, skin temperatures, and psychophysical ratings of thermal pain sensitivity across 4 series within 3 separate tests of a relatively homogeneous sample of IBS patients. The participants with clinical pain were females with IBS and diarrhea, and they were free from medications that can confound studies of a chronic pain condition. These characteristics should reduce the variability and diversity that is inherent to most chronic pain conditions but is problematic for correlational studies. For example, skin temperature of females is highly responsive to sympathetic vasoconstriction, a component of stress reactions which are affected by antidepressants often prescribed for chronic pain [23]. Clinical pain intensity was rated thoroughly and at the beginning of each test session so that it could be related concurrently to autonomic reactivity and to somatic pain sensitivity. This is important, because clinical pain intensity changes over time in different patterns for different patients. Psychophysical testing incorporated a method of repetitive thermal stimulation which evaluated pain sensitivity as the temperatures required to maintain the same average level of pain for control subjects and IBS patients. Matching of elicited pain across subjects is ideal when the intent is to generate input of comparable affective intensity to central stress circuits.

Even though the group of IBS patients reported low to moderate levels of chronic visceral pain, baseline skin temperatures were not significantly different for IBS and control subjects. However, resting skin temperature is not controlled entirely by sympathetic tone. Responsivity to environmental temperature by local vascular mechanisms can compensate for a dysregulation of tonic sympathetic outflow [42, 43]. As an alternative to measurement of resting temperature, the magnitude of skin temperature decrements in response to nociceptive stimulation can reveal tonic levels of sympathetic activation, depending upon environmental temperature and the subjects' gender [14, 25, 38]. The key to this physiological assay of sympathetic responsivity/tone is to set the test conditions for stress-free subjects so that a robust decrease in skin temperature occurs during prolonged nociceptive stimulation. Given this responsivity for control subjects, relatively high levels of tonic sympathetic activation for IBS patients were revealed by a substantial reduction in skin temperature change (by an average of 80%) contralateral to the hand receiving nociceptive stimulation. This finding indicates that chronic IBS pain was associated with tonic sympathetic vasoconstriction, which interfered with phasic sympathetic activation during nociceptive stimulation. Similar relationships between resting and elicited sympathetic activity of IBS patients have been noted for heart rate variability [12].

Despite a lack of between-group differences in resting skin temperatures, there was a significant correlation between resting skin temperatures and thermode temperatures during nociceptive testing of IBS patients. These measures were not significantly correlated for control subjects, indicating that resting temperature was regulated by different means for control and IBS subjects. Sympathetic tone appears to have determined resting skin temperatures to a greater extent for IBS patients than for controls. The relationship between skin temperatures and pain sensitivity for IBS patients corroborates the between-group difference in skin temperature change observed during nociceptive thermal stimulation. These observations suggest that high levels of tonic sympathetic tone for IBS patients induce thermal hyperalgesia, elaborating upon previous demonstrations of cutaneous hyperalgesia for IBS patients [2, 10, 27, 44]. However, levels of IBS pain were not significantly correlated with resting skin temperatures, skin temperature responses to nociceptive stimulation, or thermal pain sensitivity. Thus, elevated sympathetic tone is associated overall with chronic IBS pain, but day-to-day variations in IBS pain magnitude for the cohort of patients in this study were not dependent upon levels of sympathetic tone or its influence on pain sensitivity. 

The vicious cycle of IBS pain and sympathetic dysregulation can be conceptualized in several ways, depending on etiology: (1) stress-gut pathology and IBS pain-more stress-more pain, and so forth; or (2) gut pathology and IBS pain-stress-more pain-more stress, and so forth. Given these reciprocal interactions, it is difficult to determine the cause of IBS pain, and it may not be a simple case of either gut pathology or stress. For example, inflammation and stress could interact to produce gut pathology [7, 25, 45]. Often, however, psychological conditions such as anxiety and depression have been proposed as the initiating cause of IBS. Relevant to this issue, psychological history has been found to be unrelated to development of IBS [46]. In other studies, correlations between psychological stress and concurrent autonomic dysregulation or IBS pain have been nonexistent or weak [3, 19, 47]. These findings and the present investigation cast doubt on the proposition that psychological stress and an associated autonomic dysregulation determine IBS pain intensity. Also countering the psychogenic hypothesis, there is considerable evidence that abnormal afferent input from the gut originates and maintains IBS pain [2].

If IBS pain results from gut pathology and sympathetic dysregulation is a consequence of psychological reactions to that pain and its implications for the individual, this has important therapeutic implications. A cure for IBS pain is expected to depend upon treatments that address gut pathology. In the absence of effective gastrointestinal treatments of IBS, reduction of stress and associated hyperalgesia should be beneficial for management of pain.

## Figures and Tables

**Table 1:** Mean and (SD) for palm temperature by group.

Time 1, Baseline	Time 2, Immediately after testing	Time 3, 3-minutes after testing (recovery)
Stimulated right palm		

32.4 (1.0)	38.9 (1.0)	33.2 (1.6)
32.2 (1.1)	38.8 (1.3)	32.3 (0.8)

Nonstimulated left palm		

32.4 (0.9)	32.1 (0.9)	32.0 (0.8)
32.2 (0.9)	30.7 (0.6)	31.2 (0.6)

**Table 2:** Mean temperature (SD) during each series for IBS and controls.

Rating of 35%	Series 1 (SI = 1.0)	Series 2 (SI = 0.8)	Series 3 (SI = 1.0)	Series 4 (SI = 0.8)
48.6 (1.0)	47.5 (0.9)	46.5 (0.9)	46.0 (0.9)	46.4 (0.9)
51.6 (1.0)	50.7 (0.8)	51.0 (0.8)	50.0 (0.8)	50.5 (0.8)
3.0	3.2	4.5	4.0	4.1
